# A Comparison of Children's Ability to Read Children's and Adults' Mental States in an Adaptation of the Reading the Mind in the Eyes Task

**DOI:** 10.3389/fpsyg.2017.00594

**Published:** 2017-04-26

**Authors:** Anna van der Meulen, Simone Roerig, Doret de Ruyter, Pol van Lier, Lydia Krabbendam

**Affiliations:** ^1^Section of Clinical Developmental Psychology and Research Institute LEARN!, Faculty of Behavioural and Movement Sciences, Vrije Universiteit AmsterdamAmsterdam, Netherlands; ^2^Section of Research and Theory in Education and Research Institute LEARN!, Faculty of Behavioural and Movement Sciences, Vrije Universiteit AmsterdamAmsterdam, Netherlands; ^3^Department of Pedagogical and Educational Sciences, Faculty of Social Sciences, Erasmus University RotterdamRotterdam, Netherlands

**Keywords:** mental state reading, reading the mind in the eyes, contextual embeddedness, children's daily life, theory of mind

## Abstract

The ability to read mental states from subtle facial cues is an important part of Theory of Mind, which can contribute to children's daily life social functioning. Mental state reading performance is influenced by the specific interactions in which it is applied; familiarity with characteristics of these interactions (such as the person) can enhance performance. The aim of this research is to gain insight in this context effect for mental state reading in children, assessed with the Reading the Mind in the Eyes (RME) task that originally consists of pictures of adults' eyes. Because of differences between children and adults in roles, development and frequency of interaction, children are more familiar with mental state reading of other children. It can therefore be expected that children's mental state reading depends on whether this is assessed with children's or adults' eyes. A new 14 item version of the RME for children was constructed with pictures of children instead of adults (study 1). This task was used and compared to the original child RME in 6–10 year olds (*N* = 718, study 2) and 8–14 year olds (*N* = 182, study 3). Children in both groups performed better on the new RME than on the original RME. Item level findings of the new RME were in line with previous findings on the task and test re-test reliability (in a subgroup of older children, *n* = 95) was adequate (0.47). This suggests that the RME with children's eyes can assess children's daily life mental state reading and supplement existing ToM tasks.

## Introduction

Being able to determine the emotional or mental state of others based on subtle physical or facial cues is an important part of social cognition (Vellante et al., 2013), which in turn is important to function adequately in social environments (Heyes and Frith, 2014; Slaughter et al., 2015). This skill is often assessed with the Reading the Mind in the Eyes (RME) task. The RME consists of pictures of eyes that depict mental states. Participants are required to choose the correct mental state out of four words accompanying the picture. The task has been developed from a precursor version (Baron-Cohen et al., 1996, 1997) to its current version for adults (Baron-Cohen et al., 2001a) and for children (Baron-Cohen et al., 2001b).

Contexts such as the cultural setting in which an interaction takes place can influence mental state reading, emotion recognition and other Theory of Mind or social-cognitive abilities (Elfenbein and Ambady, 2003; Sternglanz and DePaulo, 2004; Adams et al., 2010; Calvo et al., 2014). Specifically for mental state reading this has for example resulted in an adapted version of the RME for adults with an Asian background, in which the mostly Caucasian eyes of the original version have been replaced by Asian eyes (Adams et al., 2010). In the current paper we consider how this context effect of the setting in which an interaction takes place applies to the specific situation of mental state reading and its measurement in children. The aim is to both theoretically examine how children's everyday context can affect their mental state reading and to explore this context effect by evaluating an adapted version of the Reading the Mind in the Eyes. We start with a brief description of the construct measured by the RME as well as an evaluation of the current version of the task for both adults and children.

### Construct and evaluation of the RME

In the introduction of the RME in its final form, the task was described as measuring the overall ability to attribute mental states to oneself or to another person (Baron-Cohen et al., 2001a). The RME can therefore be defined as a mental state reading or recognition task (Sabbagh, 2004; Sabbagh et al., 2004; Bora et al., 2006). Mental state reading refers to “the ability to decode mental states on the basis of immediately available information such as facial expression or tone of voice,” which is perceived as a part of Theory of Mind (Bora et al., 2006, p. 96). This ability includes both decoding basic emotions and more complex states, such as identifying whether someone is serious based on subtle facial cues (or for example detecting sarcasm in a tone of voice). This description of the RME has not been consistently used however, and the use of different definitions of the task and the construct it measures has contributed to confusion and criticism on the RME (Johnston et al., 2008; Fernández-Abascal et al., 2013). Two other criticisms that have been raised on the RME are a lack of differentiating between posed and genuine emotions in the task and the reliance upon static instead of dynamic facial expressions (Johnston et al., 2008). Although these difficulties raise serious points, a detailed analysis is beyond the scope of the current paper. Furthermore, to a certain extent previous criticisms on the RME seem related to an inadequate definition of the construct it measures.

Both the original RME and adaptations of the task (for example in different languages) have been evaluated first by assessing the task at the level of individual items. The quality and difficulty of the individual items provide an indication of the discriminative properties of the task (Fernández-Abascal et al., 2013). Variation in difficulty between items is desirable, however items of which the correct answer rate lies below 40–50% or above 90% are usually considered too difficult or too easy. Several recently translated versions of the RME for adults indicate that an average correct answer rate between 67 and 75% is normal (Hallerbäck et al., 2009; Yildirim et al., 2011; Fernández-Abascal et al., 2013; Vellante et al., 2013; Prevost et al., 2014). Unfortunately only a few reports exist of information at the item level on the child RME; in the construction of the child version (Baron-Cohen et al., 2001b), in a Swedish adult sample who completed the translated child version (Hallerbäck et al., 2009) and in a Turkish sample (Girli, 2014). A second evaluation point of the RME concerns its reliability. Test-rest reliability appears the best approach for a task such as the RME where it is only possible to compare correct answers (Fernández-Abascal et al., 2013). Generally adequate test-retest reliabilities in the form of Intraclass Correlation Coefficients have been found over several time periods (ranging from one week to one year, Yildirim et al., 2011; Fernández-Abascal et al., 2013; Vellante et al., 2013; Prevost et al., 2014). Though less suited for the RME, low to moderate Cronbachs alpha's as a measure of internal consistency have been reported as well (Vellante et al., 2013; Girli, 2014; Prevost et al., 2014). Not all reports of the RME include a measure of reliability (for example Baron-Cohen et al., 2001a,b; Dorris et al., 2004; Spek et al., 2010).

Finally, validity of the RME has not always been thoroughly considered. A higher performance of females is the most consistent finding on the RME in the non-clinical population (Vellante et al., 2013), in line with a gender difference in favor of females in related constructs such as empathy (Christov-Moore et al., 2014) and emotion understanding (Cutting and Dunn, 1999). However, the association between the RME and gender as well as the association between the RME and other ToM abilities, empathy and IQ have not been consistently found (Baron-Cohen et al., 2001a; Brent et al., 2004; Spek et al., 2010; Vellante et al., 2013). This could be due to the strong difference in content, design, and target population between these previous studies (Prevost et al., 2014). More consistently affirmed is the relation between other ToM constructs and indicators of social functioning in children such as peer popularity (Diesendruck and Ben-Eliyahu, 2006), peer social skills and social behavior (Peterson et al., 2015) and conversational skills (De Rosnay et al., 2014). These previous findings provide starting points to assess the validity of the RME by focusing on indicators of social functioning. Finally, an increase in RME performance of children from six to approximately 12 years old has been consistently shown (Baron-Cohen et al., 2001b; Girli, 2014).

### Reading mental states in specific interactions

Insights from different research lines have shown that the meaning of mental states, including simple and complex emotions, is embedded in the setting, or context, in which interactions take place (Feldman Barrett et al., 2011). One aspect of the context on which mental state reading can depend has been referred to as “the cultural context in which perceivers and targets operate” (Feldman Barrett et al., 2011). A well-documented effect of the cultural context, which refers here to ethnic background or country, is the cultural in-group advantage or same race effect. Cultures differ in the exact way emotions and mental states are expressed, and consequently individuals show an advantage in recognizing emotions and mental states in their cultural in-group (Elfenbein and Ambady, 2003; Adams et al., 2010). A similar effect has been found for the recognition of emotions in friends or partners compared to strangers (Sternglanz and DePaulo, 2004). The same mechanisms are thought to underlie these effects: a higher level of experience, practice and knowledge with individuals who are closer results in an advantage when applying mental state or emotion reading skills in interactions with these individuals. Therefore this aspect of the setting in which interactions take place can be conceptualized broader, as the “relational context.” The relational context includes but is not limited to cultural perceiver-target relationships.

Further, continuing on the mechanisms of the relational context effect, two crucial elements are familiarity and frequency. First, *familiarity* with contextual aspects of interactions (characteristics of the person with whom one interacts, emotional content of the interaction, the interaction itself in which these skills are used) enhances mental state recognition. This mechanism has been described for cultural in-groups, where exposure to the expressive behavior of someone's own group makes a person most skilled at recognizing these expressions that furthermore match his or her own style (Elfenbein and Ambady, 2003). Second, mental state recognition is further enhanced by *frequency* of exposure to these contextual aspects of interactions. Although frequency clearly has a positive influence on familiarity, frequency of exposure also has a direct effect. This is confirmed by the finding that specific emotions that occur more often in social interactions are better recognized than less frequently occurring emotions (Calvo et al., 2014).

These mechanisms of the relational context effect have specific consequences for mental state reading of children and its measurement. To measure their mental state reading with the RME, children read adults' eyes. From the perspective of the relational context however, for children reading adults' eyes is different than reading children's eyes. *First*, the mechanism of familiarity is related to the difference between child-child and adult-child relationships. Because of socialization and societal processes, adults and children have different roles. Adults are for example parents or care takers who raise the child or teachers who facilitate classroom functioning (Epstein, 1998; Bjerke, 2011). In addition, mental states of children are also more familiar to children in the sense that they match their own style of expression and are therefore more accessible to them. Furthermore, adults are more experienced in expressing and recognizing emotions and mental states and have a better understanding of “more sophisticated aspects” (Del Giudice and Colle, 2007; p. 796) of these abilities, such as suppression or faking of emotions (Del Giudice and Colle, 2007). Related to the roles they can have, adults might also be more inclined to use these subtle emotional display strategies. Therefore, because of differing roles and sophisticated emotional displays, children's expectations and representations of adult mental states differ from those of child mental states (Epstein, 1998; Christensen, 2004; Fitneva, 2010). *Second*, reading mental states of other children is not only more familiar for children (in both senses), it also occurs more frequently. In their daily life children spend a considerable amount of time interacting with, often only one or a few, other children.

The nature of the relationship between adults and children, the specific roles adults have and the developmental difference in emotion or mental state expression, together with the higher frequency of one on one interactions between children, implies that for a child reading facial cues of children is different, less ambiguous and more familiar than reading facial cues of adults. Consequently, whether the persons whose eyes are depicted in the pictures of the RME are children or adults is a contextual aspect of the task which can influence the performance of children when measuring mental state reading. Mental state reading as children engage in most in daily life might therefore be more accurately approached with an adapted version of the original child RME in which the pictures of adult eyes are replaced by children's eyes.

### Current research overview

The aim of the current research is to gain insight in the role of the relational context in mental state reading in children and its measurement using the (RME) task. A new version of the child RME is constructed in which the adult eyes are replaced by children's eyes (study 1). This new task is evaluated in a group of 6 to 10 year old children (study 2) and a group of 8 to 14 year old children (study 3). The approach of the construction of the original adult and child RME (Baron-Cohen et al., 2001a,b) and of the version with Asian eyes (Adams et al., 2010) is followed. In both study two and three the evaluation of the new RME includes a direct comparison to the original RME. Overall scores, item level answer distributions and test-retest reliabilities in sub samples of both age groups are taken into account. In order to explore the validity of the new RME, associations with gender, age, peer popularity and prosocial behavior according to peers are assessed for both the new and the original RME. Peer popularity and peer rated prosocial behavior are commonly used to assess the relation between children's social functioning and social cognition (Diesendruck and Ben-Eliyahu, 2006; Peterson et al., 2015). They seem therefore valuable potential indicators of the validity of the RME. The focus on the comparison with the original RME has been previously suggested as the best strategy when evaluating an adapted version of the RME (Prevost et al., 2014). In addition, in the current research the comparison serves the purpose of confirming that reading children's eyes is different for children than reading adults' eyes. The 6 to 10 year olds are the youngest for whom the child RME was originally considered suited (Baron-Cohen et al., 2001b), the 8–14 year olds can be considered the next successive age group.

It is expected that the performance on the new and original task differs and that children score higher on the new RME than on the original RME. Further, the new RME is expected to be reliable in the form of stable over time. Finally, a higher performance of females and a positive relation with age is expected for both the new and the original RME. A positive relation between peer popularity and prosocial behavior are expected for both the new and original RME as well. However, since peer popularity and prosocial behavior (according to peers) explicitly concern the context of children, it can be expected that reading mental states of children is most (directly) beneficial for these aspects of social functioning. Therefore, the relation between peer popularity, prosocial behavior and the new RME is expected to be stronger than the relation between peer popularity, prosocial behavior and the original RME.

## Study 1: construction new RME

### Materials and methods

#### Participants

Twenty-four young children (13 girls) from one elementary school, grade 4 participated. The children were between 6 and 8 years old during the first measurement (*M* = 7.04, *SD* = 0.36), and between 7 and 8 years during the second measurement (*M* = 7.25, *SD* = 0.44). The elementary school was located in an urban area in the Netherlands. Although data on ethnic background were not collected for this sample, study 3 included several schools from the same area and assessed ethnic background. Based on this comparison, it can be expected that between 50 and 75% of the children in the current study had a Caucasian background, and the remaining children a non-Caucasian background. It can be assumed that all children in this year of regular elementary school, as well as the equally or higher years of the second and third study, are sufficiently fluent in Dutch to be able to participate.

#### Procedure

Data were collected as part of a larger study on social developments and school achievements of elementary school children. One class of this project participated in the two rounds of the current study. Before the first round, parents received an information letter through the school and gave consent for the participation of their child. The data collection took place during school hours. Children for whom no informed consent was obtained from the parents worked quietly for themselves. Participating children read a child information letter together with the researcher and gave consent for their own participation. Children were seated apart and were told to work individually. The procedures used in the first and other studies were approved by the medical and ethical committee at VU University Medical Center.

The instruction to the RME was derived from the report of the original child RME as well as the instructions included in this instrument (Baron-Cohen et al., 2001b). This instruction was presented to the children of the second and third study as well. It states that children were to see pictures of eyes accompanied by four words, and that they had to look carefully and pick the word that best described what the person in the picture was thinking or feeling. The example item was completed first, together with the researcher. Because of the young age of the children, children were encouraged to raise their hand and ask the researcher when they did not understand a word. The researcher clarified words by describing them, giving a synonym and/or using the word in a sentence. This procedure was considered more suited for a young age group than the original procedure by Baron-Cohen where participants are presented with a glossary of all target and foil words (Baron-Cohen et al., 2001a). In addition, the researcher and children went through the task together item by item. The researcher attempted to check carefully whether all children filled out their answer before moving on to the next item. The completion of the task lasted 30–40 min. Three months later the same children participated in the second round, following the same procedure of the task.

#### Materials

The original child version of the RME consists of 28 items and has been derived from the 36 item original adult RME by changing some of the more complex mental state words to simpler words and replacing three pictures (Baron-Cohen et al., 2001a,b). The new child RME of the current study was based on these 28 combinations of targets and foils. These words were translated in Dutch by two Dutch researchers and back translated by a bilingual Dutch-English researcher.

**Figure 1:**
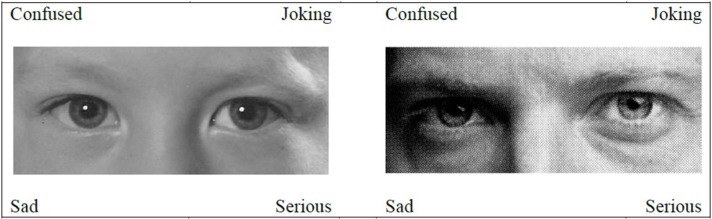
**Example items for the new and original RME**.

In all steps of the construction of the new child RME, the construction of the original RME and previous adaptations was closely followed (Baron-Cohen et al., 1997; Adams et al., 2010). Five involved researchers collected pictures of children's faces through their social network. Photos were selected of children between 6 and 12 years old showing a variety of emotional and mental states. These pictures were all adapted into black and white colors and the eye region was selected, consisting of a rectangular area of 5 by 12.5 cm around the eyes, starting above the eyebrows and ending halfway through the nose. Next, these pictures of children's eye region were matched to the 28 target mental states in open discussion between the five researchers who had collected the pictures. For each mental state the five researchers considered and compared different pictures, until all agreed on a match. The resulted task was completed by the children in the first round. Pictures were approved after this first round when at least 60% of the children choose the target mental state. This resulted in 12 approved pictures. For the second round, the remaining 16 pictures that had not been approved were replaced with new pictures matching the target words. The same procedure was used of selecting and matching pictures in an open discussion between the five researchers. This resulted in a second version of the new child RME, consisting of the 12 approved pictures of the first round and 16 new pictures. The children completed the entire task, including the pictures that had already been approved. Again, pictures were approved when at least 60% of the children chose the target mental state, which resulted in two additional approved pictures. The 14 items approved in these two rounds formed the new child RME task (from now on referred to as “new RME”) (Appendix A, Table A1). Similar to the faces in the original RME, most depicted children had a Caucasian background. Figure 1 shows an example of an item both in its version for the new RME and as it was included in the original child RME.

Children answered two background questions, on gender and age. Data from all studies were analyzed with the Statistical Package for the Social Sciences (SPSS), version 20.

### Results

Scores on the 14 item new RME as completed in the second round were normally distributed, *M* = 10.76 (*SD* = 1.62). Table 1 shows the answer distributions for all items. Correct answers were chosen by between 48 and 96% of the children (*M* = 76.86%). Two items for which the target word was chosen by more than 60% of the children in the first round, were chosen correctly during the second round by 56% (*worried*, 71% in the first round) and 48% (*thinking about something sad*, 83% in the first round) of the children. Of the three items answered correctly by more than 90% of the children (*serious, a bit worried*, and *happy*) in the second round, two items (*serious, a bit worried*) had also been answered correctly by more than 90% of the children in the first round. An independent *t*-test (assumption of equal variances not violated) showed that there were no gender differences (girls *M* = 11.00, *SD* = 1.29, boys *M* = 10.73, *SD* = 1.85), *t*_(22)_ = 0.42, *p* = 0.676, Cohen's *d* = 0.17.

**Table 1:** **Study 1 answer distributions per item for new RME (in percentage)**.

**Target mental state**	**Target**	**Foil 1**	**Foil 2**	**Foil 3**
**NEW RME**				
Sad	84	4	12	–
Friendly	88	–	8	4
Worried	56	4	16	24
Remembering	72	12	12	4
Thinking about something	72	12	–	16
Serious	96	–	–	4
Thinking about something	80	16	–	4
Not believing	80	–	16	4
A bit worried	92	–	8	–
Thinking about something sad	48	8	28	16
Not pleased	76	–	12	12
Sure about something	60	36	4	–
Happy	92	–	4	4
Scared	80	16	4	–

### Discussion

The aim of this first study was to construct a new child RME in which the pictures of adult eyes were replaced by children's eyes. Following the procedure by Baron-Cohen et al. (2001a,b) and Adams et al. (2010) 14 new pictures matching the original target words were selected and approved. This is half the length of the original 28 item child version of the RME, however this length seems suited and sufficient for a child task which should be appropriate from the age of seven. Furthermore, the resulting 14 pictures show sufficient variation in terms of difficulty (with a few items only approved by 50–60 percent of the children).

Although the original versions of the RME (Baron-Cohen et al., 2001a,b) were closely followed in the construction of and instruction in the new task, because of the young age of the children two modifications were made. First, instead of receiving a glossary explaining all target and foil mental states, the children were encouraged to ask the researcher about words they did not know. Although this approach contains the risk of children being afraid to speak up, children appeared very comfortable to enquire to the researcher. Second, the researcher and children went through the task together, item by item, instead of having the children complete the task by themselves. It is likely that these adjustments added to the length of the completion of the task, which was relatively long. Although the researcher was careful to ensure that all children understood the task, individual administration would be preferable for this age group.

Compared to the construction (Baron-Cohen et al., 2001a) and previous adaptations of the original RME (for example Prevost et al., 2014) this study had a small sample size (but see Adams et al., 2010). A next step therefore is to evaluate and validate the new task in a larger sample, in direct comparison to the original task.

## Study 2: original and new RME in 6-10 year old children

### Materials and methods

#### Participants

Children from 17 schools and 56 classes (*N* = 718; 343 girls, 374 boys, 1 gender missing) participated in study 2. The children were from grade 4 (342 children, 171 girls) or 5 (376 children, 171 girls, 1 gender missing) and were between 6 and 9 years old (*M* = 8.16, *SD* = 0.77) (2 age missing). The schools were both from an urban and more rural area in the Netherlands. Ethnic background was assessed at a later wave of the project and therefore not obtained for 205 children. For the children for whom ethnic background was known, the majority had a Caucasian background (424 children, 83% of the children for whom ethnic background was known). The remaining 89 children had a non-Caucasian background (including mostly children with a Turkish, Moroccan and Surinam background).

A subgroup of these children of study 2 (*N* = 253, 118 girls and 135 boys) had already completed the original RME in the previous school year, approximately one year earlier. Time between the first and second measurement was nine to 14 months (*M* = 11.4, *SD* = 1.07). These children were in grade 4 (24 classes) during the first measurement where they completed the original RME, all but six children had passed to grade 5 during the second measurement during which they completed both the original and new RME (28 classes). The children in the subsample were between 7 and 10 years old both during t1 (*M* = 7.64, *SD* = 0.58, 2 age missing) and t2 (*M* = 8.63, *SD* = 0.57, 1 age missing). In the subsample as well the majority of children had a Caucasian background (139 children, 85%).

#### Procedure

Data were collected as part of an ongoing longitudinal project on psychosocial development of young elementary school children. Study 2 was part of the fourth wave of the project. The subgroup of children who completed the original RME a year earlier did so during the third wave of the project. The procedure during both waves was the same. Parents were approached through the schools with an information letter and were asked for approval for their child's participation. Data collection took place during school days. Children completed the tasks and questionnaires, including the RME, during two individual sessions with trained research assistants, which took place in the morning and the afternoon. The new and original RME were combined in one task (preceded by one instruction). At the end of their participation in all tasks and questionnaires of the project the children received a small gift. The schools were debriefed both after the third and fourth wave of the data collection on the complete project.

The same instruction to the RME was given as in study 1. Similar to study 1, children did not receive a glossary with all target and foil words but were encouraged to ask after words they did not know. Completion of the new and original RME together lasted 5–10 min. Participation in only the original RME lasted 5 min.

#### Materials

Children completed the 14 item new RME constructed in study 1 combined with a short version of the original 28 item child RME (Baron-Cohen et al., 2001b) consisting of 14 items (from now on referred to as “original RME”). Prior to the construction of the new RME in study 1, this 14 item original RME was selected as a version more appropriate in length for young children (compared to the original 28 items) (Appendix A, Table A1). Items in this original version were selected to represent both simpler emotions (*sad*) and more complex cognitive states (*remembering*). Since this version had already been constructed and only during study 1 approved items were included in the new RME, eight out of the 14 items matched between these two versions (Table A1, Appendix A).

In a within subjects design all children completed the new and the original RME, 28 pictures in total, in a single session. In order to control for task order effects, half of the children started with the new RME and half of the children started with the original RME. During the precursor study children only completed the 14 items of the original child RME. All children started with the same example item.

Information on age and gender was obtained through the schools and complemented by two questions presented to the children. Further, children were asked about country of birth of their mother and father to assess ethnic background. Finally, peer nominations were used to assess children's peer popularity and prosocial behavior. Children were presented with a list of all children in the class and asked to nominate children they liked (peer popularity) and children who are nice to other children (prosocial behavior). Peer popularity and prosocial behavior were defined as the total amount of nominations a child received for this construct by classmates, divided by the class size minus one (because a child could not nominate him or herself). Popularity and prosocial behavior scores therefore ranged from 0 to 1.

### Results

#### Comparison new and original RME

Scores on the new and original RME as well as the difference scores were normally distributed. A paired *t*-test showed that children scored higher on the new RME (*M* = 8.28, *SD* = 2.29) than on the original RME (*M* = 7.09, *SD* = 2.15), *t*_(717)_ = 12.59, *p* < 0.001, Cohen's *d* = 0.47. Scores on the new and the original RME were positively related, *r*_(717)_ = 0.351, *p* < 0.001. Table 2 shows the answer distributions for both the new and original RME. For the new RME correct answers were chosen by between 43.9 and 79.8% of the children (*M* = 59.14%). Four items were answered correctly by less than 50% but more than 40% of the children (*worried, not pleased, sure about something, scared*). For the original RME, correct answers were chosen by between 14.1 and 77.7% of the children (*M* = 50.64%). Seven items were answered correctly by less than 50% *(interested, sad, friendly, worried, interested, not believing, made up her mind*), two of which had an extremely low correct answer rate (*sad*, 27.9% and *friendly*, 14.1%).

**Table 2:** **Study 2 answer distributions per item for the new and original RME (in percentage)**.

**Target mental state**	**Target**	**Foil 1**	**Foil 2**	**Foil 3**
**NEW RME**				
Sad	72.3	2.2	7.0	18.5
Friendly	79.8	2.2	13.0	4.9
Worried	45.0	9.6	15.9	29.4
Remembering	50.3	25.1	17.3	7.4
Thinking …	58.4	17.5	3.9	20.1
Serious	72.8	15.5	6.4	5.2
Thinking …	52.5	19.9	19.5	8.1
Not believing	57.4	5.8	20.9	15.9
A bit worried	67.3	12.1	12.5	8.1
Thinking …sad	71.2	3.2	18.1	7.4
Not pleased	43.9	9.5	31.9	14.8
Sure…	48.2	36.5	9.3	5.3
Happy	60.0	15.3	3.2	21.3
Scared	48.9	32.5	15.2	3.5
**ORIGINAL RME**				
Interested	42.4	22.1	29.7	5.7
Sad	27.9	29.7	32.2	10.3
Friendly	14.1	27.3	15.5	43.2
Upset	65.3	21.0	3.3	10.3
Serious	73.1	16.3	5.2	5.4
Worried	44.8	6.4	9.7	38.9
Interested	40.8	32.5	18.9	7.8
Remembering	77.7	1.1	2.9	18.2
Thinking.	70.2	15.0	7.8	6.8
Not believing	54.2	13.9	23.1	8.8
Thinking.	50.0	14.5	18.2	17.1
Not believing	44.2	6.0	11.0	38.9
Made up her mind	48.6	4.2	15.6	31.5
A bit worried	55.7	21.3	7.2	15.7

The comparison of performance on the new and original RME was repeated using only the 8 matching items of both versions, to check whether item selection affected this comparison. A paired *t*-test showed that here as well, children scored higher on the 8 item new RME (*M* = 4.76, *SD* = 1.61) than on the 8 item original RME (*M* = 4.44, *SD* = 1.54), *t*_(717)_ = 4.62, *p* < 0.001, Cohen's *d* = 0.17.

An independent *t*-test (equal variances) showed that there were no gender differences on the new RME, *t*_(715)_ = 1.21, *p* = 0.228, Cohen's *d* = 0.09 (girls: *M* = 8.39, *SD* = 2.25, boys: *M* = 8.18, *SD* = 2.32). On the original RME an independent *t*-test (equal variances) showed that girls (*M* = 7.32, *SD* = 2.16) scored significantly higher than boys (*M* = 6.87, *SD* = 2.12), *t*_(715)_ = 2.86, *p* = 0.004, Cohen's *d* = 0.21. On both RME's there was a positive association between performance and age, but for the new RME this was not significant, *r*_(714)_ = 0.068, *p* = 0.070 (*n* = 716). For the original RME the correlation between test score and age was significant, *r*_(714)_ = 0.126, *p* = 0.001 (*n* = 716). Relations between peer popularity and both new RME performance, *r*_(714)_ = 0.082, *p* = 0.028, and original RME performance, *r*_(714)_ = 0.079, *p* = 0.035, were small but significant. Prosocial behavior as well was positively related to new RME performance, *r*_(714)_ = 0.143, *p* < 0.001 and original RME performance, *r*_(714)_ = 0.130, *p* < 0.001.

To test whether there was an effect of order in which the RME's were completed, an ANOVA was conducted with RME version (2 levels; new and original) as within subjects factor and order (2 levels; new version first (*n* = 368) and original version first (*n* = 350)) as between subjects factor. The results showed the main effect of RME version but no main effect of order, *F*_(1, 716)_ = 2.87, *p* = 0.091.There was an interaction effect between RME version and order, *F*_(1, 716)_ = 54.89, *p* < 0.001, partial η^2^ = 0.07. Simple main effects analysis confirmed that although scores were higher on the new version both when started with the new version (new: *M* = 8.72, *SE* = 0.12, original: *M* = 6.87, *SE* = 0.11, *p* < 0.001, partial η^2^ = 0.039) and when started with the original version (new: *M* = 7.81, *SE* = 0.12, original: *M* = 7.32, *SE* = 0.12, *p* = 0.006, partial η^2^ = 0.011), this difference was larger when children started with the new version.

#### Test-retest original RME

Test-retest reliability was assessed for the subgroup of 253 children who already completed the original RME. The original version was completed first during the second testing time by 111 children, 142 children started with the new version. A paired *t*-test showed that performance on the original RME did not differ between the precursor study (t1) (*M* = 7.30, *SD* = 2.00) and study 2 (t2) (*M* = 7.31, *SD* = 2.09), *t*_(252)_ = −0.104, *p* = 0.917. Distributions of scores were normal and similar for both testing times. Intraclass Correlation Coefficient (ICC), single score one way model, was used as an indication for test-retest stability for the total score. ICC is used to measure the relation between two variables that measure the same construct and can therefore be used to describe the consistency of repeated measures (Field, 2005; Vellante et al., 2013). For the original RME in the current sample ICC was.301. Test-retest reliability was further assessed at the item level. Table 3 shows the answer distributions for t1 and t2 and Table 4 shows percentages of children for each item who answered “twice correct,” “twice wrong,” or “once correct and once wrong.” The combination of information in these two tables (following Prevost et al., 2014 and Yildirim et al., 2011) makes clear both whether items are answered correctly by an equal number of children across testing times (Table 3) and whether these are the same children (Table 4). Six items were answered correctly by less than 50% of the children both on t1 and t2 (*sad, friendly, worried, interested, not believing, made up her mind*, Table 3). Furthermore, five of these items were not consistently answered (by more than 50%) either right or wrong (Table 4). *Friendly*, which had an extremely low correct response rate during both testing times, was the only item which was answered consistently wrong. In addition to these six items, four other items were not answered consistently either right or wrong (*interested, not believing, thinking about something, a bit worried*, Table 4). For these items the target word was chosen by the majority of the children, although for *interested, thinking about something* not on both times (Table 3).

**Table 3:** **Study 2 answer distributions per item for t1 and t2 for original RME**.

**Target mental state**	**Target**		**Foil 1**		**Foil 2**		**Foil 3**	
	**Test**	**Retest**	**Test**	**Retest**	**Test**	**Retest**	**Test**	**Retest**
**ORIGINAL RME**								
Interested	50.6	45.8	15.8	20.6	27.3	28.5	6.3	4.7
Sad	40.3	28.1	24.1	30.0	24.1	32.4	11.5	9.5
Friendly	16.2	15.8	21.7	24.5	17.4	14.2	44.7	45.5
Upset	77.1	74.7	12.6	16.2	1.2	2.4	9.1	6.7
Serious	73.5	75.1	18.2	13.8	4.0	5.9	4.3	5.1
Worried	40.7	48.2	7.1	4.7	5.1	9.9	46.6	36.8
Interested	40.7	40.7	30.0	38.3	18.6	17.8	10.7	3.2
Remembering	69.6	81.0	0.8	1.2	1.6	2.4	28.1	15.4
Thinking.	71.1	67.2	15.0	17.4	5.9	8.3	7.9	6.7
Not believing	54.9	55.3	10.3	13.0	26.9	25.3	7.9	6.3
Thinking.	51.4	47.8	14.2	18.2	16.6	17.0	17.8	16.6
Not believing	35.6	44.7	9.1	5.1	8.3	9.9	46.6	40.3
Made up her mind	44.3	49.0	5.9	2.4	19.4	16.6	30.4	32.0
A bit worried	63.6	57.7	17.8	20.6	4.0	7.1	14.6	14.6

**Table 4:** **Study 2 percentage of participants who chose wrong twice, correct twice or once correct and once wrong**.

**Target mentalstate**	**Both wrong**	**One correct, one wrong**	**Both correct**	**Same response (right/wrong)**
**ORIGINAL RME**				
Interested	28.9	45.8	25.3	54.2
Sad	46.2	39.1	14.6	60.8
Friendly	71.9	24.1	4.0	75.9
Upset	9.9	28.5	61.7	71.6
Serious	8.7	34.0	57.3	66
Worried	30.4	50.2	19.4	49.8
Interested	37.9	42.7	19.4	57.3
Remembering	9.5	30.4	60.1	69.6
Thinking.	11.9	37.9	50.2	62.1
Not believing	22.5	44.7	32.8	55.3
Thinking.	28.9	43.1	28.1	57
Not believing	41.1	37.5	21.3	62.4
Made up her mind	30.8	45.1	24.1	54.9
A bit worried	19.4	39.9	40.7	60.1

### Discussion

In the second study of this research, the new child RME as well as the original child RME were completed by a group of young children. As expected, children performed better on the new than on the original RME. Both tasks appeared relatively difficult for the children, as can be seen in the average correct answer rates, which (with 59.14% for the new and 50.64% for the original RME) are low compared to previous adult RME reports. However, the original RME not only had the lowest average but also included a large proportion of items answered correctly by less than 50% of the children, some of which were answered correctly by less than 30%. Further, test-retest reliability in the form of ICC was 0.301 for the original RME. This can be considered poor test-retest reliability (Cicchetti, 1994). The pattern of the stability of the individual items reflects this, with both several of the easier and more difficult items answered not consistently right or wrong. Finally, as was expected on the original RME girls outperformed boys and there was a positive relation between age and performance. Contrary to the expectations however no gender difference or relation to age was found for the new RME (although for age a trend in the expected direction could be seen). Both the new and the original RME were positively but weakly related to peer popularity and prosocial behavior.

As expected, children show an advantage in reading children's eyes compared to reading adult eyes. Performance on the new RME was lower than expected, possibly because for this young age group the task is relatively difficult. The task does however show sufficient variance in this young sample. This second study suggests that it is different for children to read mental states of other children than to read mental states of adults. Our exploration of the relational context in the situation of mental state reading of children predicted this, since the mechanisms of familiarity and frequency imply that for children reading eyes of adults is more difficult and ambiguous than reading eyes of children. Though reading both eyes appeared relatively difficult for this age group, reading mental states of adults was considerably more difficult. A next step is to explore whether these differences between reading mental states of adults and mental states of children, as measured with the original child RME and the new child RME, hold in an older age group.

## Study 3: original and new RME in 8-14 year children

### Method

#### Participants

Study 3 comprised 182 children (75 girls). The children were from five elementary schools, with 25 children from grade 6, 26 children from grade 7, 20 children from grade 7/8 and 57 children from grade 8. The remaining 54 children were from two first classes of high school. Children were between 8 and 14 years old (*M* = 10.93, *SD* = 1.19). The schools were from urban areas in the Netherlands. 139 children had a Caucasian ethnic background (76%), the majority of the children with a non-Caucasian background reported they or their parents had been born in Turkey, Morocco, the Dutch Antilles or Aruba.

Approximately half a year later four elementary schools of study 3 participated again with in total 95 children (39 girls). For three schools (73 children) there were 6 months between the date of the first and second measurement, for the fourth school (22 children) this was 7 months. The children were from grade 6 (22 children) from grade 7 (23 children) and grade 8 (50 children). Both during the first and second measurement this subgroup of children was between 8 and 12 years old (first time, *M* = 10.36, *SD* = 0.93, second time, *M* = 10.84, *SD* = 0.87). Sixtynine (73%) children from the subgroup had a Caucasian background, from the remaining 26 children the majority had a Moroccan or Turkish background.

#### Procedure

Data on the new and original RME were collected as part of a larger study on perspective taking. Depending on the preference of the schools, parents were approached with an information letter or email and gave either active (two elementary schools) or passive (three elementary schools and the high school) consent. In case of active informed consent, parents filled out a consent form and handed it in to the teacher. Additional information was given in case of passive informed consent, stating that if parents objected to participation of their child they would have to communicate this to the teacher and that all children would automatically participate if no objection by a parent was made. After receiving parental consent, children received an information letter themselves as well and gave their own consent by filling out a form or signing a list. Five months later all schools were approached for the retest. Four schools were willing and able to participate. The parents of the children who were part of this subsample were approached again through the schools with an information letter, following the same procedure (obtaining either active or passive consent depending on the school). Other questionnaires of the study besides both versions of the RME were not completed again. Only parents of the children who had already participated in the first measurement were approached, and children only participated if consent was obtained again. During both test and retest children were seated apart from each other and worked individually during testing. Children who did not participate worked quietly for themselves. Afterwards, children were thanked and received candy. All schools were debriefed after the retest.

The same instruction to the RME as in study 1 and 2 was given. Because the children in this study were older, they received a glossary with definitions of all target and foil words and sentences in which the words were used.

#### Materials

Children completed the same combination of the 14 item new and 14 original RME as in study 2 in a within subject design. After starting with the same example, half of the children started with the new child version and half of the children started with the original child version to control for task order effects.

The children answered several background questions during both testing times, including on gender, age and country of birth of their mother and father to assess ethnic background.

### Results

#### Comparison new and original RME

The distribution of the difference scores as well as the distribution of scores on the new RME showed kurtosis values of 2.93 (for the differences scores), 2.25 (for t1), and 1.58 (for t2). Scores on the original RME were normally distributed. A paired *t*-test showed that children scored higher on the new RME (*M* = 10.27, *SD* = 1.94) than on the original RME (*M* = 8.45, *SD* = 1.94), *t*_(181)_ = 9.57, *p* < 0.001, Cohen's *d* = 0.71. There was a positive but not significant association between scores on the new and original RME, *r*_(181)_ = 0.122, *p* = 0.100. Table 5 shows the answer distributions for the new and original RME. For the new RME correct answer rates were between 47.8 and 91.2% (*M* = 73.34%). Two items were answered correctly by less than 50% but more than 40% of the children (*remembering* and *thinking about something*). One item was answered correctly by more than 90% of the children (*friendly*, 91.2%). For the original RME correct answer rates were between 16.5 and 91.2% (*M* = 60.63%). Four items were answered correctly by less than 50% of the children (*interested, friendly, interested, not believing*), two of which had an extremely low correct answer rate (*interested*, 39.6% and *friendly*, 16.5%). One items was answered correctly by more than 90% of the children (*serious*, 91.2%).

**Table 5:** **Study 3 answer distributions per item for the new and original RME (in percentage)**.

**Target mental state**	**Target**	**Foil 1**	**Foil 2**	**Foil 3**
**NEW RME**				
Sad	80.2	2.7	6.6	10.4
Friendly	91.2	1.6	4.4	2.2
Worried	48.9	8.2	14.3	28.0
Remembering	47.8	33.0	15.4	3.8
Thinking …	58.8	15.4	1.1	24.2
Serious	84.6	8.8	1.6	4.4
Thinking …	68.7	20.9	7.1	3.3
Not believing	84.6	2.2	5.5	7.7
A bit worried	85.7	3.3	9.9	0.5
Thinking …sad	76.9	1.6	17.0	4.4
Not pleased	69.2	5.5	20.3	3.8
Sure…	72.5	23.1	3.3	1.1
Happy	80.2	4.9	2.7	12.1
Scared	77.5	14.3	7.1	1.1
**ORIGINAL RME**				
Interested	39.6	39.6	14.8	6.0
Sad	49.5	22.5	23.1	4.9
Friendly	16.5	35.2	9.9	37.9
Upset	87.4	9.9	1.6	1.1
Serious	91.2	6.6	-	2.2
Worried	54.9	2.7	2.2	39.6
Interested	49.5	20.3	20.3	9.3
Remembering	86.3	2.2	2.7	8.8
Thinking.	72.5	14.8	9.3	3.3
Not believing	58.2	15.4	19.8	6.6
Thinking.	65.9	22.0	3.8	8.2
Not believing	49.5	1.1	5.5	43.4
Made up her mind	73.6	1.6	10.4	13.7
A bit worried	50.5	26.9	4.9	17.0

The comparison of performance on the new and original RME was repeated using only the 8 matching items of both versions, to check whether item selection affected this comparison. A paired *t*-test confirmed that children scored higher on the 8 item new RME (*M* = 5.59, *SD* = 1.33) than on the 8 item original RME (*M* = 5.20, *SD* = 1.50), *t*_(181)_ = 42.70, *p* = 0.008, Cohen's *d* = 0.20.

An independent *t*-test (equal variances) showed no gender differences on the new RME (girls *M* = 10.47, *SD* = 2.01, boys *M* = 10.13, *SD* = 1.88), *t*_(180)_ = −1.15, *p* = 0.250, Cohen's *d* = 0.17. On the original RME as well, performance of girls and boys did not differ (girls *M* = 8.44, *SD* = 2.02, boys *M* = 8.46, *SD* = 1.89), *t*_(180)_ = 0.061, *p* = 0.951, Cohen's *d* = −0.01. Performance on both the new and original RME was positively related to age, this relation was only significant for the new RME, *r*_(180)_ = 0.151, *p* = 0.042. For the original RME the relation between age and performance was not significant, *r*_(180)_ = 0.021, *p* = 0.775. In order to fully assess the relation with age, all children from the young (study 2) and older (study 3) age group who completed both the new and original RME were taken together (*N* = 898). In this combined sample age was positively related to both the new RME, *r*_(896)_ = 0.319, *p* < 0.001, and to the original RME, *r*_(896)_ = 0.257, *p* < 0.001.

As in study 2, to test whether there was an order effect, an ANOVA was conducted with RME version (2 levels; new and original) as within subjects factor and order (2 levels; new version first (*n* = 96) and original version first (*n* = 86)) as between subjects factor. The results showed the main effect of version but no main effect of order, *F*_(1, 180)_ = 0.661, *p* = 0.417. Here as well an interaction effect between RME version and order was present, *F*_(1, 180)_ = 19.34, *p* < 0.001, partial η^2^ = 0.097. As in study 2, simple main effects analysis confirmed higher scores on the new version when started with that version (new: *M* = 10.73, *SE* = 0.19, original: *M* = 8.16, *SE* = 0.20, *p* < 0.001, partial η^2^ = 0.371) and when started with the original version (new: *M* = 9.76, *SE* = 0.20, original: *M* = 8.78, SE = 0.21, *p* < 0.001, partial η^2^ = 0.071). Mean scores and effect sizes show that the difference between the version was larger when children started with the new RME.

#### Test-retest original and new RME

Test-retest reliability was assessed for both RME's in the subsample of 95 children. More than half (52) of these children started with the same version during the first and second measurement (new version: 27 children, original version: 25 children). The remaining 43 children either completed the new version first during the first measurement and the original version first during the second measurement (*n* = 22) or vice versa (*n* = 21). A paired *t*-test showed that for the new RME scores did not differ between t1 (*M* = 10.17, *SD* = 2.03) and t2 (*M* = 10.35, *SD* = 1.88), *t*_(94)_ = −0.86, *p* = 0.390. On the original RME as well scores did not differ between t1 (*M* = 8.63, *SD* = 2.09) and t2 (*M* = 8.83, *SD* = 1.93), *t*_(94)_ = −0.81, *p* = 0.420. Distributions of the scores were similar for both test times for both tasks, for the new task the distributions were pointy. As in study 2, test-retest stability for the total score was assessed with Intraclass Correlation Coefficient (ICC), single score one way model. For the new RME test-retest stability assessed with the ICC was 0.469, for the original RME ICC was 0.288. Test-retest reliability was assessed for both RME's at the item level by looking at answer distributions at t1 and t2 (Table 6) and percentages of children who answered consistently right, wrong or inconsistently (Table 7). First, for the new RME one item (*remembering*) was answered correctly by less than 50% (but more than 40%) on both t1 and t2 (Table 6), this item was also not consistently answered right or wrong (Table 7). Three additional items were not answered consistently right or wrong by the majority of children (*worried, thinking about something*, and *thinking about something*, Table 7). These items were answered correctly however by more than 50% on both testing times (*worried* by 48.4% on t1). Second, for the original RME, two items were answered correctly by less than 50% of the children (*interested* and *friendly*, Table 6). *Friendly* was answered consistently wrong by the majority of the children, *interested* however was not answered consistently right or wrong. Seven additional items were not answered consistently right or wrong by the majority of the children (*sad, worried, interested, not believing, thinking about something, not believing, worried*). These items did however have a higher than 50% correct answer rate at both testing times.

**Table 6:** **Study 3 answer distributions per item for t1 and t2 for new and original RME**.

**Target mental state**	**Target**		**Foil 1**		**Foil 2**		**Foil 3**	
	**Test**	**Retest**	**Test**	**Retest**	**Test**	**Retest**	**Test**	**Retest**
**NEW RME**								
Sad	84.2	80.0	4.2	–	4.2	8.4	7.4	11.6
Friendly	88.4	93.7	3.2	2.1	4.2	3.2	3.2	–
Worried	48.4	52.6	9.5	13.7	13.7	11.6	28.4	21.1
Remembering	47.4	46.3	30.5	27.4	17.9	15.8	4.2	10.5
Thinking …	57.9	68.4	15.8	7.4	1.1	5.3	24.2	18.9
Serious	82.1	83.2	13.7	9.5	1.1	2.1	3.2	4.2
Thinking …	69.5	62.1	22.1	29.5	4.2	3.2	4.2	4.2
Not believing	88.4	82.1	1.1	–	2.1	13.7	8.4	3.2
A bit worried	88.4	87.4	4.2	3.2	7.4	7.4	–	2.1
Thinking …sad	75.8	76.8	2.1	3.2	17.9	17.9	4.2	7.4
Not pleased	67.4	72.6	5.3	–	22.1	24.2	4.2	2.1
Sure …	64.2	75.8	32.6	20.0	2.1	1.1	1.1	1.1
Happy	76.8	83.2	4.2	1.1	3.2	5.3	15.8	10.5
Scared	77.9	70.5	13.7	20.0	8.4	6.3	–	3.2
**ORIGINAL RME**								
Interested	41.1	42.1	34.7	36.8	17.9	13.7	6.3	6.3
Sad	58.9	54.7	16.8	22.1	20.0	18.9	4.2	4.2
Friendly	11.6	13.7	43.2	28.4	11.6	15.8	33.7	41.1
Upset	85.3	86.3	9.5	10.5	3.2	–	2.1	
Serious	93.7	94.7	5.3	2.1	–	–	1.1	1.1
Worried	63.2	60.0	2.1	6.3	3.2	9.5	31.6	23.2
Interested	51.6	57.9	18.9	16.8	22.1	18.9	7.4	5.3
Remembering	88.4	90.5	3.2	1.1	1.1	2.1	7.4	6.3
Thinking.	75.8	69.5	13.7	14.7	8.4	5.3	2.1	10.5
Not believing	56.8	56.8	12.6	17.9	23.2	15.8	7.4	7.4
Thinking.	61.1	66.3	28.4	21.1	3.2	3.2	7.4	9.5
Not believing	55.8	58.9	2.1	3.2	3.2	6.3	38.9	28.4
Made up her mind	67.4	73.7	2.1	–	11.6	6.3	17.9	20.0
A bit worried	52.6	57.9	26.3	16.8	4.2	6.3	16.8	18.9

**Table 7:** **Study 3 percentage of participants who chose wrong twice, correct twice or once correct and once wrong**.

**Target mental state**	**Both wrong**	**One correct, one wrong**	**Both correct**	**Same response (right/wrong)**
**NEW RME**				
Sad	10.5	14.7	74.7	85.2
Friendly	1.1	15.8	83.2	84.3
Worried	27.4	44.2	28.4	55.8
Remembering	32.6	41.1	26.3	58.9
Thinking …	17.9	37.9	44.2	62.1
Serious	4.2	26.3	69.5	73.7
Thinking …	14.7	38.9	46.3	61
Not believing	–	29.5	70.5	70.5
A bit worried	2.1	20.0	77.9	80
Thinking …sad	13.7	20.0	66.3	80
Not pleased	12.6	34.7	52.6	65.2
Sure …	14.7	30.5	54.7	69.4
Happy	12.6	14.7	72.6	85.2
Scared	12.6	26.3	61.1	73.7
**ORIGINAL RME**				
Interested	36.8	43.2	20.0	56.8
Sad	23.2	40.0	36.8	60
Friendly	76.8	21.1	2.1	78.9
Upset	5.3	17.9	76.8	82.1
Serious	–	11.6	88.4	88.4
Worried	15.8	45.3	38.9	54.7
Interested	29.5	31.6	38.9	68.4
Remembering	5.3	10.5	84.2	89.5
Thinking.	12.6	29.5	57.9	70.5
Not believing	23.2	40.0	36.8	60.0
Thinking.	20.0	32.6	47.4	67.4
Not believing	22.1	41.1	36.8	58.9
Made up her mind	13.7	31.6	54.7	68.4
A bit worried	26.3	36.8	36.8	63.1

### Discussion

The third study of this research included an older group of children who completed both the new and original RME. In line with the expectations and with study 2, the older children performed better on the new than on the original child RME. Comparable to study 2 as well, the original child RME appeared difficult, with an average correct answer rate of 60.63%. Again, several items appeared relatively or very difficult. For the new RME however, contrary to study 2, the average correct answer rate (73%) and pattern of correct answers was in line with previously found patterns in adult samples. ICC for the new RME, indicating test-retest reliability, was 0.469 which is usually considered moderate to fair in non-clinical samples (Cicchetti, 1994). This is confirmed by the reliability of the individual items, which are answered either consistently right or consistently wrong. For the original RME again test-retest reliability was poor, ICC was 0.288. Finally, performance of girls and boys did not differ on both the new and the original RME. Age was positively related to performance on the new RME and a positive association could be seen between age and performance on the original RME. Taking sample one and two together shows that in the overall group of children between six and 14 years old age is positively related to performance on the new and original RME.

Overall the findings in the older age group again suggest the expected relational context effect, showing that it is different for children to read children's eyes than to read adults' eyes. Furthermore, it appears that whereas reading children's eyes was still relatively difficult for the young children of study 2, older children seem capable to read children's eyes.

## General discussion

The aim of this research was to gain insight in the relational context in children's mental state reading, specifically by exploring its role in the measurement of mental state reading with the (RME) task. A new version of the child RME was constructed in which the pictures of adults were replaced by pictures of children. The new RME was evaluated in young and older children by comparing performance, task reliability and the relation with gender, age and social functioning to the original RME.

### Performance, reliability, gender, age, and social functioning on new and original RME

First, as expected children of both age groups performed better on the new RME than on the original RME. The pattern of answer distributions of the older age group confirmed that performance on the new RME was comparable to previous adult RME reports (Hallerbäck et al., 2009; Yildirim et al., 2011; Fernández-Abascal et al., 2013; Vellante et al., 2013; Prevost et al., 2014). For the younger children the task seems to be relatively difficult. In both samples however the new RME shows sufficient variance, and the inclusion of some more difficult items suggests adequate discriminative properties of the task (Hallerbäck et al., 2009). The original RME on the other hand seems too difficult in both age groups, since it includes a high amount of relatively and extremely difficult items. Compared to previous adult RME reports (Hallerbäck et al., 2009; Yildirim et al., 2011; Fernández-Abascal et al., 2013; Vellante et al., 2013; Prevost et al., 2014), the original RME in the current samples has a low average correct answer rate and a high amount of items answered correctly by less than 50% of the children (especially in the young age group).

Children scored consistently higher on the new RME, whether they started with that version or with the original version. For the children who started with the new RME however the difference in performance between both tasks was larger. It could be that the items of the new RME were, partly since they were easier, experienced as more accessible and more motivating which increased performance especially when this was the first experience with the overall combined task. At the same time, performance on the original RME might have decreased when this version was presented second because of tiredness combined with being demotivated by the more difficult items.

Second, test-retest reliability in the form of the ICC (computed only for the older children) was fair (0.469), but lower than previously found ICC's for the adult RME, which are all above 0.6 (Hallerbäck et al., 2009; Yildirim et al., 2011; Fernández-Abascal et al., 2013; Vellante et al., 2013; Prevost et al., 2014). There are no previous reports of ICC for the original child RME, which makes it difficult to determine whether the acceptable but lower stability in our sample might be related to the age of the participants. The ICC for the original RME in our sample was poor in both the young and older children (Cicchetti, 1994). Because of the counterbalancing of order, children did not consistently start with the new or the original RME during the first and second measurement. Although this can have created noise in the obtained stability for the two tasks, it is likely that this resulted in an underestimation of the stability of the tasks.

Finally, for both RME versions the relation with gender, age and social functioning (peer popularity and peer rated prosocial behavior) was assessed. First, with the new RME no gender differences were found, although girls tended to score slightly higher. On the original RME as well no gender differences were found in the older sample, in the younger sample girls performed better. Although it was expected that girls would perform higher than boys based on the previously reported gender difference in favor of females in mental state reading in both adults (Vellante et al., 2013) and children (Dorris et al., 2004; Girli, 2014), it has been observed previously that this gender difference is not consistent (Vellante et al., 2013) and was not found in the study in which the original child RME was constructed (Baron-Cohen et al., 2001b). Second, in the overall group of children between 6 and 14 years old, age is positively related to performance on both the new and original RME, in line with our expectation and previous findings (Baron-Cohen et al., 2001b; Girli, 2014). For the new RME this same trend can be seen within the two age samples, although the effects are small and only significant in the older sample. Only in the younger age group a small age effect was present for the original RME. Interestingly, performance on the new RME in the first study was relatively high compared to similar age groups of the second study, which could be because the children of the first study only completed pictures with eyes of children and not pictures with adults' eyes of the original RME as well. As the effects of task order suggest, completing pictures with children's eyes might have a motivating effect. Peer popularity and prosocial behavior were related to both the new and original RME. These effects were however very small, especially for peer popularity. Moreover, there were no differences between the new and original RME in effect size for both indicators of social functioning.

### Familiarity and frequency in findings new and original RME

It was expected that children differ in their ability to read children's and adults' mental states because of relational context effects, which would be most apparent in their higher ability to read children's eyes. This difference was confirmed by the comparison between scores on the new and original RME. The persistence of this difference in the comparison between the eight items that match exactly in terms of target word and foils but differ only in their use of children or adult eyes affirms that the higher performance on the new RME was due to the difference between children's and adults' eyes in the tasks and not to the difference in item selection. In addition, both the distribution of correct answer rates and the adequate test re-test stability suggest that the new RME is an appropriate task to measure children's daily mental state reading. This is most evident in the older children. In the younger children the task requires further investigation, specifically concerning its stability. Finally, effects for gender, age and peer rated popularity and prosocial behavior do not differ between the new and original RME. Assessing the relation with these factors does not only provide starting points for assessing the validity of the new task, but can also be used to gain insight in how these factors might be differently related to reading children's and adults' eyes. First, the inconsistency in both our and previous findings for the relation between gender and Theory of Mind abilities is likely to be related to other factors previously found to be involved, such as language or IQ (Brent et al., 2004; Engel et al., 2014). So far no conclusion can be drawn on gender differences in mental state reading of either children or adults or on a difference between these abilities regarding gender. Second, the findings on age suggest that performance on both mental state reading of children's and adults' eyes improves in childhood. This improvement is gradual however, and as the test re test findings show as well might not or not clearly be present over the course of one or a few years. Third, the relations between peer popularity as well prosocial behavior and the new RME were weak, and it is likely that the large sample size of study two contributed to establishing these significant associations. However, a recent meta-analysis confirmed that associations between task measures of Theory of Mind and indicators of social functioning often are small (Imuta et al., 2016). Considering the difficulty in establishing validity specifically for the RME, (Prevost et al., 2014), the current findings are meaningful since they indicate the potential value of these factors to establish validity of the new RME. Finally, although a positive relation was expected between the indicators of social functioning and both RME tasks, contrary to our expectation these relations were not stronger for the new RME than for the original RME. Future studies could explore how different indicators of social functioning relate to mental state reading of children's eyes and of adults' eyes.

### Other aspects of daily interactions

With this adaptation of the RME we have been exploring how our theoretical elaboration of the relational context can apply to children's mental state reading. The notion of a higher familiarity of reading children's mental states is grounded in a wider view on context effects on mental state reading: the general theory that emotional meanings are always to some extent dependent on characteristics of the setting or context in which interactions take place (Feldman Barrett et al., 2011). This implies that several other aspects of the relational context, besides the basic child-adult distinction, can be applied to children's reading of mental states. First, the relational context which impacts mental state reading refers to characteristics of perceivers and targets, which in children includes more than just the child-adult feature. Research with adults shows familiarity effects of ethnic backgrounds and perceived closeness (friends compared to strangers) (Elfenbein and Ambady, 2003; Sternglanz and DePaulo, 2004; Adams et al., 2010) on emotion recognition. Furthermore, findings on development of emotion, mental state perception or Theory of Mind emphasize that children show different developmental trajectories for ToM skills depending on the cultural context in which they grow up (Slaughter and Perez-Zapata, 2014). These cultural contexts effects also involve familiarity with or frequency of exposure to talking about emotions and internal mental states. Children with various cultural backgrounds can differ in this aspect because of cultural practices, such as whether it is common to emphasize mental states as an explanation of behavior (Slaughter and Perez-Zapata, 2014). Second, the child-adult feature itself can also include other characteristics which can impact mental state reading. Although the idea that adults and children and their mutual relationships differ can be said to be generally universal, strong differences exist per society and culture in the exact roles adults and children take, the extent to which these roles are fixed, the extent to which more sophisticated aspects of emotional displays are used by adults in these roles and the difference in frequency of interaction (Epstein, 1998; Bjerke, 2011).

These additions to the relational context effect we explore can be taken into account when interpreting RME findings and can for some specific situations suggest other adaptations. For example, studying mental state reading in Asian children might be approached with an Asian child version of the RME, taking into account two relational contexts effects: the child-adult distinction and the Western-Asian distinction (Adams et al., 2010).

### The new RME as a research instrument and limitations

By exploring the effect of the relational context on children's mental state reading, we constructed the new child RME which has the potential for a new instrument that measures children's daily life mental state reading. The value of such an instrument can be found in the current emphasis on a broad and multi-facet approach in ToM research in children (Wellman and Liu, 2004; Shahaeian et al., 2011; Tahiroglu et al., 2014). It has been stressed that other aspects of ToM functioning should be included besides the traditionally most studied false beliefs (Wellman and Liu, 2004) as well as multiple informants, contexts and instruments (Tahiroglu et al., 2014). The new child RME can contribute to this broader approach by detecting meaningful differences in one aspect of ToM closer to children's everyday context. The importance of adequate ToM functioning of children has been shown for several aspects of their daily life social functioning, including their conversational skills (De Rosnay et al., 2014), their peer social skills (Peterson et al., 2015) and the quality of their peer relationships (Slaughter et al., 2015). Individual differences in the different facets of ToM is therefore valuable knowledge that could be used as a starting point for interventions that improve Theory of Mind, thereby improving children's social functioning, adaptation and well-being (Slaughter et al., 2015).

The current research contains some limitations. First, our evaluation of the new RME focused primarily on the comparison with the original RME in overall performance and psychometric properties. Although a valuable first step in the evaluation of the new task (Prevost et al., 2014), a further assessment of its validity, exceeding our exploration on gender, age, and social functioning, is necessary. Previous findings on associations between the RME and other ToM abilities, empathy or IQ are inconsistent and difficult to build on, but this could be related to confusion on the construct of the RME as well as confounding context effects. A next step in validating the new RME could therefore be to more carefully assess the relation between children's daily life mental state reading and other aspects of their daily life social functioning involving other children and adults. Second, one important limitation of the RME task in general concerns its use of static instead of dynamic facial expressions (Johnston et al., 2008; Krumhuber et al., 2013). Though it has been common in emotion research to rely upon static images of emotional expressions, there is clear evidence for the, beneficial, role of dynamic features (Krumhuber et al., 2013). It can be considered in future studies whether the use of dynamic faces can further enhance the ecological validity of the RME. Third, for the comparison between performance on the new and original RME it would have been most preferable if the tasks had consisted of the exact same items. However, the replication of the higher performance on the new RME using only the eight matching items strongly suggests that it was not the item selection but the inclusion of child instead of adult faces that increased performance on the new RME. Fourth, a possible way to further assess whether the new RME is easier because of the child context effect and not because of the inclusion of easier items, is by having a group of adults complete both the version with children's eyes and the version with adults' eyes. This might be an interesting next step in itself, since it addresses adults' ability to read both children's and adults' eyes. Even though adults interact more frequently with adults and might therefore be more familiar with reading adults' eyes, it could be that adults prove equally skilled at reading both eyes or even better at reading children's eyes because of the developmental difference between child's and adults' expressions. This is related to a remark on the value of the original RME with adults' eyes in the population of children. This task can be used in children as well, however it should be understood that it measures specifically their ability to read mental states of adults.

Concluding, the new child RME explores how the relational context affects children's mental state reading. The value of this paper lies first in its consideration of this context effect for the situation of children, and second in its presentation of a potentially valuable addition to the battery of ToM related tasks for children that incorporates this effect.

## Ethics statement

This study was carried out in accordance with the recommendations of “The medical and ethical committee at the VU University Medical Center” with written informed consent from all subjects. All subjects gave written informed consent in accordance with the Declaration of Helsinki. The protocol was approved by the “The medical and ethical committee at the VU University Medical Center.”

## Author contributions

Av and SR developed the concept of the research. The organization of the data collection was done primarily by Av, in close collaboration with SR, and together with and supervised by Pv (study 2) and Dd and LK (study 1, 2, and 3). Av analyzed the data, assisted and guided by SR, Dd, and LK. Av, SR, Dd, and LK interpreted the results. After the initial draft by Av and SR, Av wrote the manuscript. SR, Dd, and LK provided critical comments on the manuscript at several stages, Pv provided comments at the last stage of the manuscript. All authors approved the final version of the manuscript for publication.

### Conflict of interest statement

The authors declare that the research was conducted in the absence of any commercial or financial relationships that could be construed as a potential conflict of interest.
