# Supplementary material for: A Comparison of Children's Ability to Read Children's and Adults' Mental States in an Adaptation of the Reading the Mind in the Eyes Task
Source: Front Psychol. 2017 Apr 26;8:594. doi: 10.3389/fpsyg.2017.00594 (PMC5405343; doi:10.3389/fpsyg.2017.00594)
Supplement: Supplementary file 1 [file DataSheet1.docx]

**Appendix A**

Table A1

*Target and foil words for the new and original child RME.*

| Item | Target answer | Foil 1 | Foil 2 | Foil 3 |
| --- | --- | --- | --- | --- |
| New RME | | | | |
| *1* | *Sad** | *Unkind* | *Cross* | *Surprised* |
| *2* | *Friendly* | *Sad* | *Surprised* | *Worried* |
| *3* | *Worried** | *Hate* | *Unkind* | *Bored* |
| *4* | *Remembering** | *Happy* | *Friendly* | *Angry* |
| *5* | *Thinking about something** | *Annoyed* | *Hate* | *Surprised* |
| *6* | *Serious** | *Confused* | *Joking* | *Sad* |
| *7* | *Thinking about something** | *Upset* | *Excited* | *Happy* |
| *8* | *Not believing** | *Friendly* | *Wanting to play* | *Relaxed* |
| *9* | *A bit worried** | *Angry* | *Friendly* | *Unkind* |
| *10* | *Thinking about something sad* | *Angry* | *Bossy* | *Friendly* |
| *11* | *Not pleased* | *Kind* | *Surprised* | *Excited* |
| *12* | *Sure about something* | *Surprised* | *Joking* | *Happy* |
| *13* | *Happy* | *Disgust* | *Hate* | *Bored* |
| *14* | *Scared* | *Jealous* | *Relaxed* | *Hate* |
| Original RME | | | | |
| *1* | *Interested* | *Angry* | *Daydreaming* | *Sad* |
| *2* | *Sad** | *Unkind* | *Cross* | *Surprised* |
| *3* | *Friendly* | *Sad* | *Surprised* | *Worried* |
| *4* | *Upset* | *Relaxed* | *Surprised* | *Excited* |
| *5* | *Serious** | *Confused* | *Joking* | *Sad* |
| *6* | *Worried** | *Hate* | *Unkind* | *Bored* |
| *7* | *Interested* | *Feeling sorry* | *Bored* | *Joking* |
| *8* | *Remembering** | *Happy* | *Friendly* | *Angry* |
| *9* | *Thinking about something** | *Annoyed* | *Hate* | *Surprised* |
| *10* | *Not believing* | *Kind* | *Shy* | *Sad* |
| *11* | *Thinking about something** | *Upset* | *Excited* | *Happy* |
| *12* | *Not believing** | *Friendly* | *Wanting to play* | *Relaxed* |
| *13* | *Made up her mind* | *Joking* | *Surprised* | *Bored* |
| *14* | *A bit worried** | *Angry* | *Friendly* | *Unkind* |

*Note.* * refers to combinations of target and foil words included in both the new and the original RME.
